# Quantification of an Antibody-Conjugated Drug in Fat Plasma by an Affinity Capture LC-MS/MS Method for a Novel Prenyl Transferase-Mediated Site-Specific Antibody–Drug Conjugate

**DOI:** 10.3390/molecules25071515

**Published:** 2020-03-26

**Authors:** Byeong ill Lee, Min-Ho Park, Jin-Ju Byeon, Seok-Ho Shin, Jangmi Choi, Yuri Park, Yun-Hee Park, Jeiwook Chae, Young G. Shin

**Affiliations:** 1 College of Pharmacy and Institute of Drug Research and Development, Chungnam National University, Daejeon 34134, Korea; byungill.lee.cnu@gmail.com (B.i.L.); minho.park.cnu@gmail.com (M.-H.P.); jinju.byeon.cnu@gmail.com (J.-J.B.); seokho.shin.cnu@gmail.com (S.-H.S.); jangmi.choi.cnu@gmail.com (J.C.); yuri.park.cnu@gmail.com (Y.P.); 2 LegoChemBiosciences, Inc. 8-26 Munpyeongseo-ro Daedeok-gu Daejeon 34302, Korea; yuniya@legochembio.com (Y.-H.P.); chae@legochembio.com (J.C.)

**Keywords:** antibody-conjugated drug, LC-MS/MS, bioanalysis, antibody–drug conjugate, beta-glucuronidase

## Abstract

The novel prenyl transferase-mediated, site-specific, antibody–drug conjugate LCB14-0110 is comprised of a proprietary beta-glucuronide linker and a payload (Monomethyl auristatin F, MMAF, an inhibitor for tubulin polymerization) attached to human epidermal growth factor receptor 2 (HER2)-targeting trastuzumab. A LC-MS/MS method was developed to quantify the antibody-conjugated drug (acDrug) for in vitro linker stability and preclinical pharmacokinetic studies. The method consisted of affinity capture, enzymatic cleavage of acDrug, and LC-MS/MS analysis in the positive ion mode. A quadratic regression (weighted 1/concentration^2^), with the equation y = ax^2^ + bx + c, was used to fit calibration curves over the concentration range of 19.17~958.67 ng/mL for acDrug. The qualification run met the acceptance criteria of ±25% accuracy and precision values for quality control (QC) samples. The overall recovery was 42.61%. The dilution integrity was for a series of 5-fold dilutions with accuracy and precision values ranging within ±25%. The stability results indicated that acDrug was stable at all stability test conditions (short-term: 1 day, long-term: 10 months, Freeze/Thaw (F/T): 3 cycles). This qualified method was successfully applied to in vitro linker stability and pharmacokinetic case studies of acDrug in rats.

## 1. Introduction

The antibody–drug conjugate (ADC) technology platform uses an antibody, linkers, and chemical drugs. Chemical drugs are conjugated to an antibody by chemical linkers to deliver the chemical drugs to the target [1]. Target-specific binding of a monoclonal antibody and highly potent, cytotoxic, chemical drugs are the major characteristics of ADCs for chemotherapy [2,3]. Seven ADCs have been approved by the US Food and Drug Administration (FDA): inotuzumab emtansine (BESPONSA), ado-trastuzumab emtansine (KADCYLA), brentuximab vedotin (ADCETRIS), gemtuzumab ozogamicin (MYLOTARG), polatuzumab vedotin (POLIVY), enfortumab vedotin (PADCEV), and trastuzumab deruxtecan (ENHERTU) [4,5,6,7]. The remarkable developments on ADC platform were achieved by means of the advancement on its components, including antibody engineering, site-specific conjugation, and new cytotoxic drugs. Approximately eighty ADCs were at various clinical phases in 2019 and many more ADCs are at preclinical phases [8]. Most of them have a heterogeneous characteristic, which makes manufacturing and processing as well as the analysis of pharmacokinetic profiles very challenging. Even some deleterious (or undesired) effects (higher aggregation, faster clearance, etc.) are observed in the heterogeneous ADCs. In order to overcome limitations originating from the heterogeneity, several site-specific ADCs have recently been developed. The site-specific ADC described in this paper is LCB14-0110, an ADC drug developed by LegoChem Biosciences Inc. that develops ADC linkers and toxins via the ConjuALL™ platform technology. LCB14-0110 is currently in phase 1 clinical trials in China and is composed of a payload (MMAF, an inhibitor for tubulin polymerization) attached to HER2-targeting trastuzumab with a CaaX sequence through a novel site-specific conjugation. This novel site-specific conjugation method is comprised of the insertion of the CaaX sequence at the C-terminal of the protein binder, prenylation using farnesyltransferase, and drug conjugation through an oxime ligation reaction [9]. MMAF and trastuzumab are used as the antitumor agent and protein binder, respectively. This site-specific ADC is homogenous and stable [7]. Several bioanalytical approaches for the development of ADCs have been discussed by many researchers [10,11,12]. The bioanalysis of ADCs has many challenges with absorption, distribution, metabolism, and elimination (ADME) due to their complexity derived from three distinct components—the cytotoxic chemical drug, the linker, and the antibody [13]. There are several major analytes that can be used for the evaluation of ADCs’ pharmacokinetic (PK) profile, including free drug and its metabolites, antibody-conjugated drug (acDrug), total antibody (tAb), drug-conjugated antibody, and naked antibody [12]. Among them, acDrug is the active analyte of ADC and is also used to calculate the drug–antibody ratio (DAR) profile, which is known as one of the most critical characteristics in ADC evaluation [14,15]. Conventionally, the bioanalysis for tAb and conjugated antibody has been conducted by ligand-binding assay (LBA) [11,16,17,18,19]. In spite of the many advantages of LBA, it is often labor-intensive and assay development of LBA is occasionally challenging [16]. Therefore, it is beneficial to have complementary tools, which can also support or confirm the bioanalytical results from LBA.

In this paper, an affinity capture LC-MS/MS method for the novel prenyl transferase-mediated, site-specific, antibody-drug conjugate LCB14-0110 in rat plasma was developed for preclinical pharmacokinetic assessment. Beta-glucuronidase was used for specific cleavage of the novel prenyl transferase-mediated, site-specific conjugation, and the propriety beta-glucuronide linker attached to the antibody (trastuzumab) and the released chemical drug (MMAF) were analyzed by LC-MS/MS. This method was able to directly measure the chemical drugs linked to the antibody. The method was qualified and applied to an in vitro linker stability study and a preclinical PK study. To the authors’ best knowledge, this is the first assay for the quantification of acDrug for the novel prenyl transferase-mediated, site-specific antibody-drug conjugate using LC-MS/MS.

## 2. Results and Discussions

### 2.1. Method Development and Qualification

**Figure 1:**
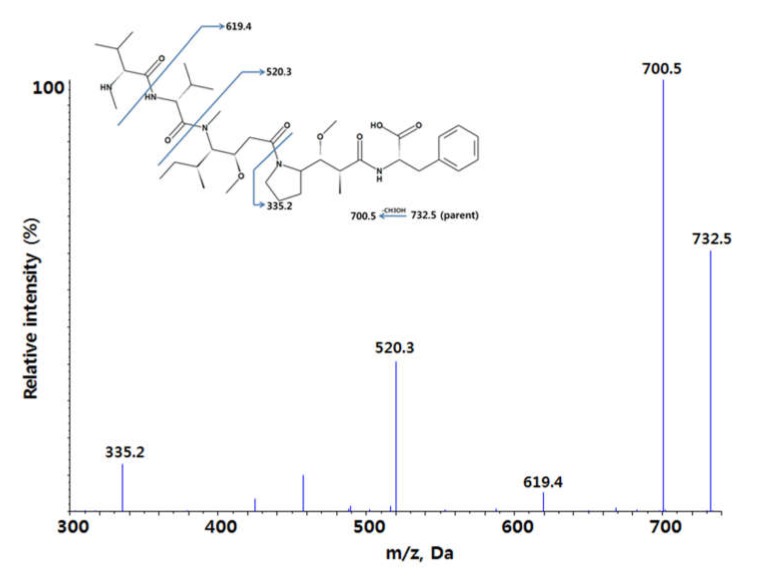
MS/MS spectrum of the antibody-conjugated drug (acDrug), MMAF.

LC-MS/MS, in the positive ion mode, was used for the quantification of acDrug (MMAF, m.w. 732.5 Da). Figure 1 shows the MS/MS spectra of acDrug (MMAF), which is a loss of CH_3_OH. The parent [M + H]^+^ ion was detected at *m*/*z* 732.5. The most abundant product ion was at *m*/*z* 700.5.

**Figure 2:**
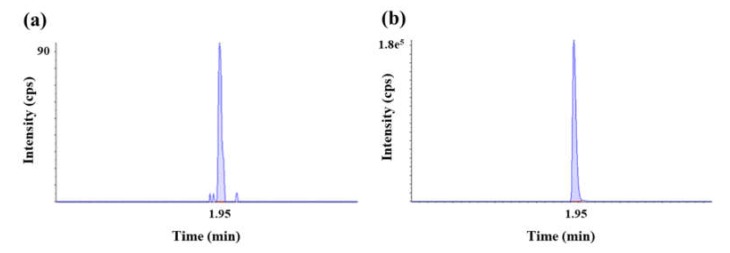
Representative chromatograms of (**a**) LLOQ (19.17 ng/mL) and (**b**) high QC (479.33 ng/mL) for acDrug (MMAF) in rat plasma.

Method qualification was carried out with a ‘fit-for-purpose’ approach. Calibration curves with seven points in duplicate were freshly prepared for all data sets. The lower limit of quantification (LLOQ) of the assay was determined to be 19.17 ng/mL. The calibration curve range was 19.17–958.67 ng/mL. Representative chromatograms of the LLOQ (19.17 ng/mL) and high QC (479.33 ng/mL) are also shown in Figure 2. 

A quadratic regression (weighted 1/concentration^2^), with the equation y = ax^2^ + bx + c, was used to fit calibration curves over the concentration range of 19.17–958.67 ng/mL for acDrug. The coefficient of determination (r) value for calibration curves of acDrug was used to evaluate the fit of the curves. The correlation coefficients of the calibration curve were greater than 0.993 for acDrug. 

Accuracy (%) and precision (% coefficient of variation (CV)) of QC samples were evaluated for examining assay performance and the results are shown in Table 1. 

The qualification run met the acceptance criteria of ±25% accuracy and precision for QC samples, which is acceptable for the early-stage drug discovery study.

**Figure 3:**
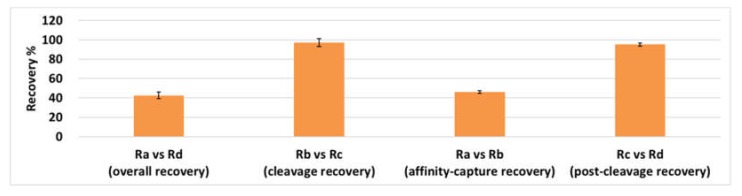
Recovery of sample preparation steps for acDrug analysis.

This quantification assay of acDrug consisted of four steps: (a) affinity capture of LCB14-0110, (b) cleavage of acDrug by beta-glucuronidase, (c) post-cleavage, and (d) LC-MS/MS analysis for the cleaved MMAF. In the recovery sample A (Ra), LCB14-0110 was added before affinity capture by magnetic beads. This sample was prepared by the standard protocol described in the Section 3.3. In the recovery sample B (Rb), LCB14-0110 was added after affinity capture by magnetic beads and before the cleavage of acDrug by beta-glucuronidase. In the recovery sample C (Rc), the MMAF concentration equivalent to LCB14-0110 molar concentration was added after affinity capture by magnetic beads and before the cleavage of acDrug by beta-glucuronidase. In the recovery sample D (Rd), the MMAF concentration equivalent to LCB14-0110 molar concentration was added after the cleavage of acDrug by beta-glucuronidase and before LC-MS/MS analysis. Affinity capture recovery by magnetic beads was determined by the ratio between Ra and Rb and the calculated value was about 45.95%. Cleavage recovery by beta-glucuronidase was determined by the ratio between Rb and Rc and the calculated value was about 97.39%. Post-cleavage recovery was determined by the ratio between Rc and Rd and the calculated value was about 95.21%. Therefore, the ratio between Ra and Rd represented the overall recovery and the calculated value was about 42.61% (done in duplicates). The recovery graph of each sample preparation steps is shown in Figure 3. 

As a result, the bottle neck of sample preparation step appeared to be the affinity capture step using magnetic beads. The total recovery could be improved if the number of magnetic beads was increased or more selective beads were used. 

The accuracy (%) and precision (%CV) of the dilution QC sample (5-fold dilution) met the acceptance criteria of ±25% (Table 2). This meant that study samples with concentrations above the upper limit of quantification (ULOQ) can be analyzed to obtain the acceptable concentration in calibration range after proper dilution with blank rat plasma.

The results for stability tests are summarized in Table 3. 

The accuracy (%) and precision (%CV) of stability samples met the acceptance criteria of ±25%. As a result, acDrug in rat plasma was stable under the different experimental conditions (short-term, long-term, and freeze/thaw).

### 2.2. Application for In Vitro Linker Stability Study and Preclinical PK Study in Rats

**Figure 4:**
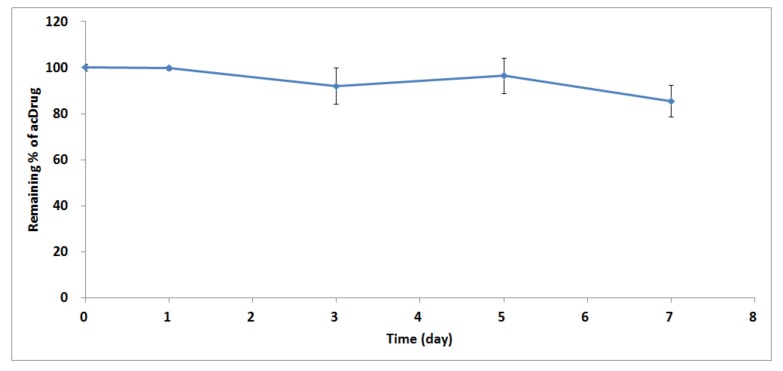
In vitro linker stability of acDrug in LCB14-0110 ADC in rat plasma.

The qualified LC-MS/MS method was applied for an in vitro linker stability study and a preclinical PK study in rats. The in vitro linker stability samples were incubated in 37 °C for 0, 1, 3, 5, and 7 days. The result is shown in Figure 4. 

About 85.38% of acDrug remained on day seven in rat plasma. Only a small amount of MMAF from LCB14-0110 was released as free payload or catabolized in rat plasma. It was suggested that this ADC was very stable in plasma and no/little payloads would be released in this experimental condition.

**Figure 5:**
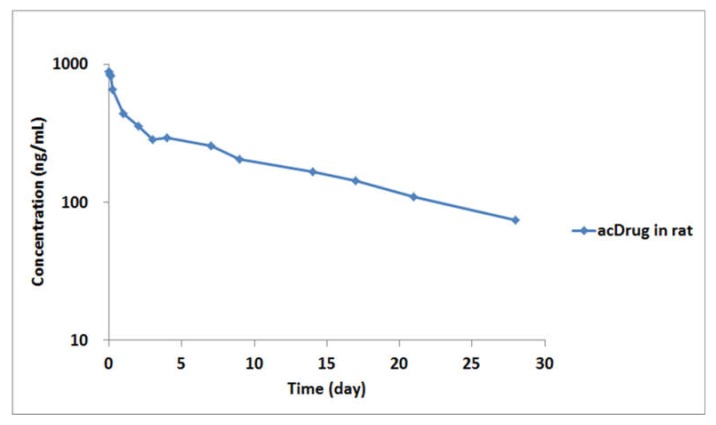
Pharmacokinetic profile of acDrug after intravenous administration of 3 mg/kg LCB14-0110 ADC in rats.

In vivo rat plasma samples obtained after intravenous administration of 3 mg/kg of LCB14-0110 were also analyzed by using this method. For acceptance of study sample analytical runs, at least two-thirds of the QC samples had to be within ±25% accuracy with at least half of the QC samples at each concentration meeting these criteria. The pharmacokinetic profile of acDrug is shown in Figure 5. 

Table 4 shows the pharmacokinetic parameters of acDrug calculated from the PK study in rats. Although preclinical pharmacokinetic study in rats was conducted at *n* = 3, total antibody (tAb) and free payload also had to be analyzed through the identical bioanalytical plasma sample obtained from each individual group, so the remaining plasma sample was pooled for the analysis of acDrug. Therefore, the standard deviation value was not expressed. Since the standard deviation from the analytical results of tAb and free payload showed very low values (data not shown), we anticipated that the standard deviation of acDrug would also be very low.

From the PK perspective, the half-life of acDrug from LCB14-0110 was similar to that of trastuzumab. It means that the linker of LCB14-0110 is very stable in systemic circulation in vivo. These results also correlated well with the in vitro linker stability study. 

The qualified LC-MS/MS method was thus successfully applied to an in vitro linker stability study and a preclinical PK study in rats.

## 3. Materials and Methods 

### 3.1. Chemicals and Reagents

**Figure 6:**
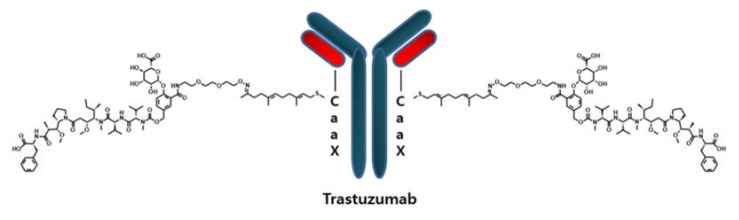
Structure of LCB14-0110 ADC.

LCB14-0110, a HER2-targeting ADC based on trastuzumab (Figure 6), was obtained from LegoChem Biosciences Inc. (Daejeon, Korea). 

Verapamil and beta-glucuronidase were purchased from Sigma-Aldrich (St. Louis, MO, USA). Formic acid was purchased from Daejung Chemical (Gyonggi-do, Korea). All other chemicals were commercial products of analytical or reagent grade and used without further purification.

### 3.2. Preparation of Stocks, Standard (STD), and Quality Control (QC) Samples

LCB14-0110 stock solutions were prepared in phosphate-buffered solution (PBS) and stored at −80 °C. The calibration curve was made by seven calibration standards at the final plasma concentration range of 19.17~958.67 ng/mL. Final plasma concentrations of QC samples were 239.67 (low QC) and 479.33 (high QC) ng/mL. Verapamil was used as the internal standard (IS).

### 3.3. Sample Preparation

**Figure 7:**
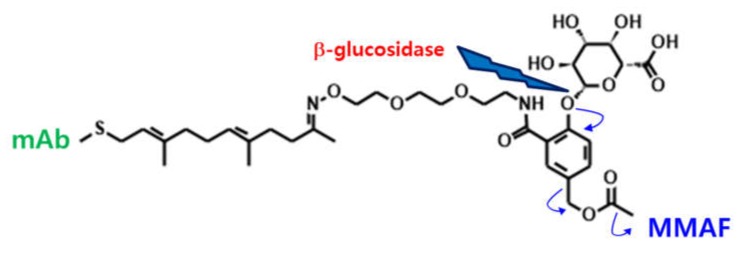
Scheme of the cleavage mechanism of site-specific enzymatic cleavage of beta-glucosidase.

Each 50 μL aliquot of study samples, QCs, and STDs were mixed with 374 μL of PBS and 30 μL of magnetic bead suspension. After gently shaking at room temperature for 2 h, the magnetic beads were washed using 600 μL of PBS, two times. Forty microliter of 1 mg/mL beta-glucuronidase in PBS was added to tubes containing the washed magnetic beads to release MMAF from LCB14-0110. Figure 7 shows the cleavage mechanism of site-specific enzymatic cleavage of beta-glucosidase. 

After shaking the mixtures for 1 min, the mixtures were incubated for 3 h at 37 °C. Forty microliter of acetonitrile containing IS was added to the mixtures to quench the activity of beta-glucuronidase. After shaking the mixtures for 1 min, the mixtures were centrifuged at 7000 rpm for 7 min and 60 μL of supernatant was mixed with 60 μL of water for LC-MS/MS analysis.

### 3.4. LC-MS/MS Conditions

The liquid chromatography–high-resolution mass spectrometric system consisted of a Shimadzu CBM-20A HPLC pump controller (Shimadzu Corporation, Columbia, MD, USA), two Shimadzu LC-20AD pumps, a CTC HTS PAL autosampler (LEAP Technologies, Carrboro, NC, USA), and a quadrupole time-of-flight TripleTOF^TM^ 5600 mass spectrometer (Sciex, Foster City, CA, USA). The HPLC analytical column used was a Kinetex XB-C18 column (2.1 × 30 mm; Phenomenex). The mobile phase consisted of: mobile phase A, distilled and deionized water containing 0.1% formic acid; and mobile phase B, acetonitrile containing 0.1% formic acid. The gradient was as follows: from 0 min to 0.5 min, 5% B; from 0.5 min to 1.7 min by a linear gradient from 5% B to 95% B; 95% B was maintained for 0.3 min; from 2.0 min to 2.1 min by a linear gradient from 95% B to 5% B; and then 5% B was maintained for 1.4 min for column re-equilibrium. The gradient was delivered at a flow rate of 0.4 mL/min and the injection volume was 10 μL.

The TOF-MS scan mass spectra and the product ion scan mass spectra were recorded in the positive ion mode. The scan range was *m*/*z* 100~800 for both TOF-MS scan and product ion scan. For the quantification, [M + H]^+^ ion of MMAF and IS (*m*/*z* 732.5 and 455.3, respectively) were selected and their product ions at *m*/*z* 700.5 and 165.1 were used for quantitative analysis, respectively. The source temperature was set at 500 °C with a curtain gas flow of 30 L/min. The ion spray voltage was set at 5500 V. For MMAF and IS, declustering potential was 100 V and 100 V, and the collision energy was 36 V and 30 V, respectively.

### 3.5. Method Qualification

Method qualification was carried out with a ‘fit-for-purpose’ approach. The qualification run contained duplicate standards at seven concentrations and QCs at two concentrations. The acceptance criteria for standards and QCs in the qualification run were within ±25% of the precision and accuracy values. Calibration was done by establishing a quadratic regression function with the equation y = ax^2^ + bx + c after 1/concentration^2^ weighting. In addition, two blank plasma samples were run. The accuracy and precision were calculated at each QC concentration.

Overall, recovery from the affinity capture, cleavage of beta-glucuronidase and the post-cleavage process of LCB14-0110 was also assessed. 

The dilution integrity test was conducted by 5 times dilution using rat plasma. The acceptance criteria for dilution integrity were within ± 25% of the precision and accuracy values. 

Stability tests were conducted in rat plasma sample under different conditions, such as short-term, long-term, and freeze/thaw stability at low and high QC. The short-term stability was determined at room temperature for 1 day. The long-term stability was determined by analyzing QC samples kept frozen at −80 °C for 10 months. For the freeze/thaw stability, the samples were subjected to three freeze-and-thaw cycles at −80 °C. The acceptance criteria for all stability tests were within ±25% of the precision and accuracy values.

### 3.6. Application for In Vitro Linker Stability Study and Preclinical PK Study in Rats

LCB14-0110 in rat plasma was used for an in vitro stability study. The samples were incubated at 37 °C for 0, 1, 3, 5, and 7 days. After incubation, the samples were stored at −80 °C before analysis. The preclinical PK study was conducted in Sprague−Dawley rats. Animal experiments followed the animal care protocol (no. CNU-00685) approved by the Chungnam National University. All procedures related to animal experiments were also performed in accordance with the guidelines established by the Association for Assessment and Accreditation of Laboratory Animal Care International (AAALAC International). LCB14-0110 was administered to rats via single intravenous bolus injection (3 mg/kg). The blood sampling times were 0, 0.002, 0.042, 0.125, 0.25, 1, 2, 3, 4, 7, 9, 14, 17, 21, and 28 days and each of the blood samples was collected in heparinated tubes. Blood samples were centrifuged and the supernatant plasma was stored at −80 °C. Pharmacokinetic calculations were performed using WinNonlin^®^ version 8.0.0 (Pharsight Corporation, Mountain View, CA, USA).

## 4. Conclusions

LCB14-0110 is a novel antibody–drug conjugate comprised of MMAF attached to HER2-targeting trastuzumab through a novel site-specific conjugation. This novel site conjugation method is comprised of the insertion of the CaaX sequence at the C-terminal of the protein binder, prenylation using farnesyltransferase, and drug conjugation through an oxime ligation reaction [9]. MMAF and trastuzumab are used as the antitumor agent and protein binder, respectively. This ADC is homogenous and stable [7].

In this paper, an affinity-capture LC-MS/MS method for LCB14-0110 in rat plasma was successfully developed and applied for in vitro linker stability and preclinical pharmacokinetic assessment in rats. Beta-glucuronidase used in this study was able to specifically cleave the novel prenyl transferase-mediated, site-specific conjugation and the propriety beta-glucuronide linker attached to the antibody. The method was qualified in a dynamic range of 19.17~958.67 ng/mL using quadratic regression with 1/concentration^2^ weighting, which was sufficient enough to cover the pharmacokinetics of LCB14-0110 acDrug in rats when administered at 3 mg/kg IV. This affinity capture LC-MS/MS method was accurate and reproducible for the determination of acDrug concentration. LCB14-0110 showed excellent PK profile with enhanced linker stability, which could potentially improve the efficacy and safety in clinical studies. The results in this paper will help studies related to ADCs with a similar linker platform. The limitation in this paper is that the affinity capture LC-MS/MS method measured the parent form of the antibody-conjugated payload, but not other catabolites simultaneously. If other catabolites were also active with a pharmacological activity as well as the parent payload, the quantitation of catabolites as well as the parent payload simultaneously would be helpful to understand the pharmacological effect of the ADC.

## Figures and Tables

**Table 1:** Quality control result for acDrug (MMAF) in rat plasma.

QC	Theoretical Concentration (ng/mL)	Mean Concentration (ng/mL)	Accuracy (%)	Precision (% CV)	*n*
Low QC	239.67	260.61	108.74	7.09	10
High QC	479.33	543.29	113.34	3.18	10

**Table 2:** The dilution integrity assessment in rat plasma.

Statistics	Dilution QC (4793.35 ng/mL)
Mean concentration (ng/mL)	5558.85
Accuracy (%)	115.97
Precision (% CV)	4.77
*n*	4

**Table 3:** The preliminary stability assessments in rat plasma.

Assessments	Theoretical Concentration (ng/mL)	Calculated Concentration (ng/mL)	Accuracy (%)	Precision (%CV)	*n*
Short-term (room temperature, 1 day)	239.67	254.72	106.28	3.21	3
	479.33	541.53	107.34	6.12	3
Long-term (−80 ℃, 10 months)	239.67	239.43	99.90	3.26	3
	479.33	541.71	113.01	4.99	3
Freeze-thaw (−80 ℃, 3 cycles)	239.67	250.69	104.6	3.58	3
	479.33	537.66	112.17	7.78	3

**Table 4:** Pharmacokinetic parameters of acDrug after intravenous (IV) administration of 3 mg/kg LCB14-0110 in rats.

Administration	AUC (ng/day/mL)	CL (mL×day/kg)	Alpha HL (day)	Beta HL (day)	C_max_ (ng/mL)	V_1_ (mL/kg)	V_ss_ (mL/kg)
Intravenous (IV)	6679.43	4.31	0.37	12.13	890.42	32.35	72.35
